# Wearable energy-smart ribbons for synchronous energy harvest and storage

**DOI:** 10.1038/ncomms13319

**Published:** 2016-11-11

**Authors:** Chao Li, Md. Monirul Islam, Julian Moore, Joseph Sleppy, Caleb Morrison, Konstantin Konstantinov, Shi Xue Dou, Chait Renduchintala, Jayan Thomas

**Affiliations:** 1 NanoScience Technology Center, University of Central Florida, Orlando, Florida 32826, USA; 2 Department of Materials Science and Engineering, University of Central Florida, Orlando, Florida 32816, USA; 3 Institute for Superconducting and Electronic Materials, University of Wollongong, North Wollongong, New South Wales 2500, Australia; 4 Institute of Simulation and Training, University of Central Florida, Orlando, Florida 32816, USA; 5 CREOL, The college of Optics and Photonics, University of Central Florida, Orlando, Florida 32816, USA

## Abstract

A promising energy source for many current and future applications is a ribbon-like device that could simultaneously harvest and store energy. Due to the high flexibility and weavable property, a fabric/matrix made using these ribbons could be highly beneficial for powering wearable electronics. Unlike the approach of using two separate devices, here we report a ribbon that integrates a solar cell and a supercapacitor. The electrons generated by the solar cell are directly transferred and stored on the reverse side of its electrode which in turn also functions as an electrode for the supercapacitor. When the flexible solar ribbon is illuminated with simulated solar light, the supercapacitor holds an energy density of 1.15 mWh cm^−3^ and a power density of 243 mW cm^−3^. Moreover, these ribbons are successfully woven into a fabric form. Our all-solid-state ribbon unveils a highly flexible and portable self-sufficient energy system with potential applications in wearables, drones and electric vehicles.

## Abstract (web summary)

Flexible materials for harvesting and storing energy are desirable for wearable electronics, but efficiency is still an issue. Here, the authors demonstrate a flexible and weavable ribbon which integrates a solar cell and supercapacitor via a shared electrode for efficient energy harvesting and storage.

## Graphical abstract

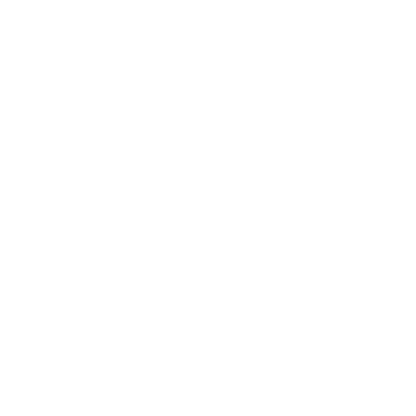

Finding efficient methods to harvest and store energy is important for current and future technological advancements^1^,^2^,^3^,^4^,^5^,^6^. Presently, most portable electronic and wearable devices carry rechargeable batteries, even when they are used in places where solar energy is plentiful. Typically, in solar powered devices, solar energy is harvested by solar cells and stored in storage devices. This is usually accomplished by two distinct devices that causes considerable space constraints when used in portable devices^7^,^8^. However, it would be highly beneficial for many portable energy applications if both functions were performed by a single device^9^,^10^. For example, currently cars parked directly under the sun fail to utilize the abundant energy from sunlight^9^,^10^. If these ribbons are incorporated into jackets, they could be used to charge wearable electronics^11^,^12^,^13^,^14^,^15^. Thus, a single ribbon that can simultaneously harvest and store electric energy would be desirable for achieving this goal.

Considerable efforts to produce integrated devices have already been endeavored by combining photoelectrodes and wet storage devices, but these methods are not suitable for wearables or other similar applications due to their device architectures^7^. Attempts to power wearables using two independent energy harvesting and storing devices have already been pursued^16^,^17^,^18^, but a single ribbon that can perform both functions is still lacking, to the best of our knowledge.

In this study, we report an all-solid-state, energy harvesting and storing (ENHANS) ribbon that integrates a perovskite solar cell (PSC) on top of a symmetric supercapacitor (SSC) via a copper (Cu) ribbon which works as a shared electrode for direct charge transfer. A highly flexible, thin, sandwich PSC with >10% conversion efficiency is developed by a solvent-assisted perovskite growth technique^19^. The sandwich-type device configuration provides better protection for the solar cell against environmental effects due to an unexposed perovskite layer to the atmosphere. The Cu ribbon not only serves as an electron-collecting electrode for the solar cell but also as a substrate for generating copper hydroxide nanotubes (CuOHNT) for developing the supercapacitor. A SSC is developed by gluing a second CuOHNT-based Cu ribbon with polyvinyl alcohol (PVA) containing potassium hydroxide (KOH) gel electrolyte onto the CuOHNT grown side of the copper ribbon electrode of the solar cell. Making use of the shared electrode, the charges generated by the flexible thin-film PSC are directly transferred and stored in the CuOHNT of the supercapacitor. Developing ENHANS on a copper ribbon may provide a low-cost solution for flexible self-sufficient energy systems for wearables and other portable devices. For example, a jacket made of this textile could harvest solar energy and directly store this energy to power wearable devices that require low-energy density or intermittent usage. These devices could also be operated continuously by alternating the power supply between adjacent sets of ENHANS using an electronic control circuit; while one set of ribbons charges, another set of ribbons discharges. We demonstrate that ENHANS ribbons can be made into a matrix/textile that may be useful for powering devices, buildings or vehicles^20^,^21^. A similar approach may also be used to develop integrated solar cells and thin-film Li-ion batteries. Since the energy density of Li-ion batteries is higher than that of supercapacitors,^22^ fabricating thin-film Li-ion batteries instead of supercapacitors on the reverse side of the solar cell ribbon would considerably improve the ribbon's energy density. By fabricating Li-ion battery–solar cell ribbons and supercapacitor–solar cell ribbons via our shared electrode approach and weaving them side by side, a matrix with high-energy density and power density may be achievable.

## Results

### Working principle of ENHANS

**Figure 1:**
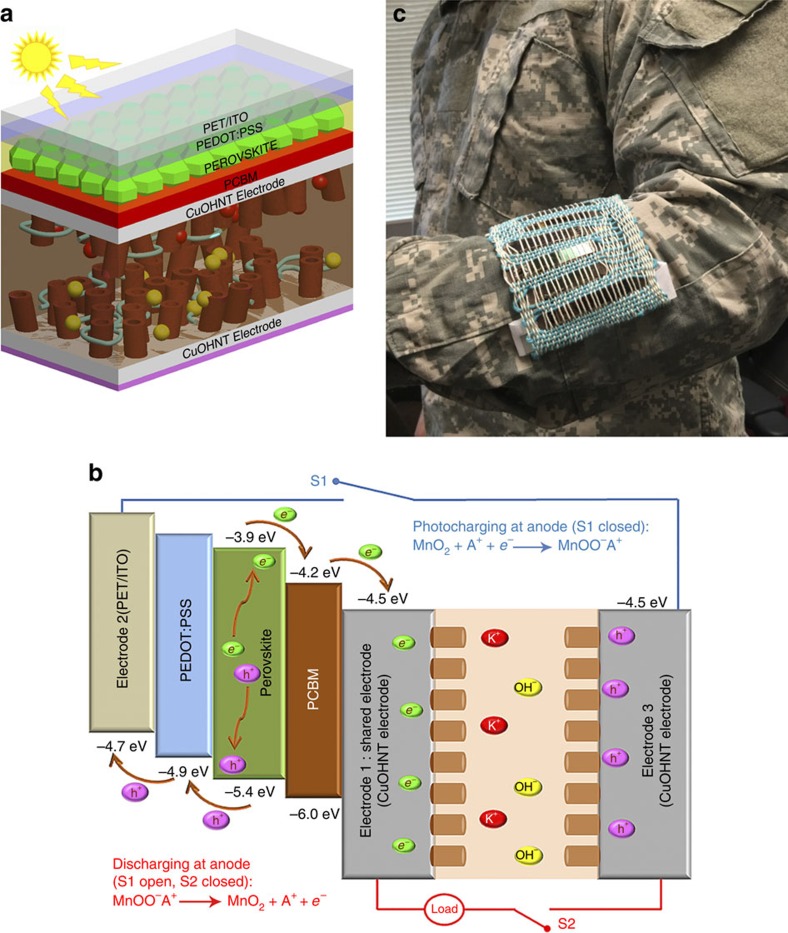
Schematic illustration and charge transfer mechanism of ENHANS ribbons. (**a**) Schematic showing ENHANS ribbon consisting of the top PSCs and bottom SSC with shared copper electrode. This ‘bi-functional energy ribbon' has been realized based on the copper ribbon as an anode (Electrode 1) for both PSCs and SSCs, while ITO/PET and another thin copper ribbon act as cathodes (Electrodes 2 and 3) for PSCs and SSC, respectively. (**b**) Charge transfer mechanism of the combination device: as ITO/PET and copper tape are connected to form close circuit (S1 closed), the photogenerated holes and electrons from PSCs flow into the cathode and anode of SSC, respectively. This current flow leads to the charging of SSC. The energy stored through the charging process can be discharged to do external work (S1 open and S2 closed). (**c**) A photograph showing a military uniform incorporating a lightweight fabric woven with ENHANS ribbons and cotton threads.

The schematic of the ENHANS ribbon architecture and charge transfer mechanism is shown in Fig. 1 to illustrate its working principle. The supercapacitor is prepared with CuOHNT electrodeposited with MnO_2_ on a Cu tape. This offers the opportunity to integrate high-performance solar-harvesting perovskite materials on the other side of the Cu electrode to transform it into a single bi-functional energy device (detailed fabrication approach is presented in Supplementary Fig. 1). Figure 1a represents the schematic of the integrated device. As shown in Fig. 1b, ENHANS ribbon consists of three electrodes: (1) negative electrode used as a shared electrode between PSC and supercapacitor; (2) positive electrode of the PSC; and (3) positive electrode of the supercapacitor. To generate charges in the PSC and successfully transfer the harvested charges to the supercapacitor for storage, the copper ribbon (electrode 1) of the supercapacitor is used as the shared electrode. One end of the positive electrode (electrode 2; top electrode) of the solar cell is directly connected to the cathode (electrode 3; bottom electrode) of the supercapacitor through a switch (Supplementary Fig. 1e). Figure 1b demonstrates the proposed electron transfer mechanism of ENHANS. During photocharging (switch S1 closed), the photoactive perovskite layer generates electrons (*e*^−^) as well as holes (h^+^), the electrons move to the electron transport layer phenyl-C61-butyric acid methyl ester (PCBM) and then to the electron-collecting electrode (copper ribbon) of the solar cell from where it is directly transferred to the anode of the supercapacitor. At the same time, holes move from the perovskite layer to the hole transport layer made up of poly(3,4-ethylenedioxythiophene) polystyrene sulfonate (PEDOT:PSS) and to the hole collector indium tin oxide (ITO) electrode of the solar cell. The electrons arrived at the anode of the supercapacitor are stored by the pseudocapacitive reaction^23^, MnO_2_+A^+^+*e*^−^→MnOO^−^A^+^, where A^+^ is either the potassium ion (K^+^) or the hydrogen ion (H^+^). Simultaneously, the reverse reaction at the cathode (MnOO^−^A^+^→MnO_2_+A^+^+*e*^−^) releases an electron. This electron moves through the outer copper foil electrode and combines with a hole at the hole collector electrode of the solar cell. Note that the top ITO electrode of the solar cell and the bottom copper ribbon electrode of the supercapacitor works like a switch (Supplementary Fig. 1e). Detailed charging and discharging reactions are given in the Supplementary Information. During the discharging process (S1 is open and S2 is closed in Fig. 1), the stored charge in the supercapacitor discharges and provides power to the connected device. A photograph showing a military uniform incorporating a light weight fabric woven with ENHANS ribbons and cotton threads is shown in Fig. 1c.

### Fabrication and performance of supercapacitor

**Figure 2:**
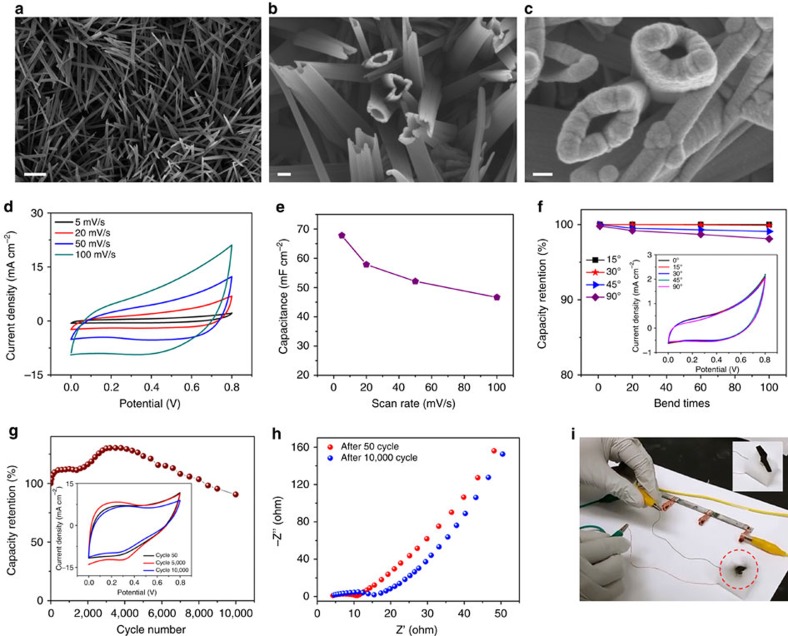
Nanoarchitecture of electrode and electrical analysis of ENHANS device. (**a**) Low (scale bar, 2 μm)- and (**b**) high (scale bar, 200 nm)-magnification FE-SEM images of CuOHNT represents the length (∼8 μm) as well as diameter (∼200 nm) of the room temperature grown CuOHNT, (**c**) high-magnification FE-SEM image of MnO_2_-deposited tubular nanoarchitecture (scale bar, 80 nm), (**d**) CV profile of the device at different scan rates, (**e**) cell capacitance calculated from the CV curves, (**f**) capacitance of the device at different bending angles (inset shows the CV curves at various bending angle), (**g**) cycle life study to explore the continuous electrochemical effect on the stability of the MnO_2_-deposited nanotubes (inset represents the CV curve of the device at certain cycle numbers), (**h**) Nyquist plot obtained from the EIS study of the as-prepared device and (**i**) rotating a propeller of toy drone with three charged supercapacitors in series. Inset is the picture of the propeller before connecting to the supercapacitor.

We developed a binder-free nanostructured ribbon supercapacitor that can be easily integrated with current and future energy generation technologies. This was accomplished by growing CuOHNT of ∼200 nm diameter and ∼10 μm in length, on a commercially available copper ribbon (Supplementary Fig. 2). In brief, CuOHNT are grown by a 20 min room temperature wet chemical process^24^,^25^,^26^. The scanning electron microscope (SEM) images in Fig. 2a,b represent the hollow tubular structure of the grown CuOHNT. The low- and high-resolution transmission electron microscope (TEM) images also serve as a clear evidence of the hollow nature of the nanotubes (Supplementary Fig. 3).

To characterize the capacitive behaviour of the electrode, we have performed cyclic voltammetry (CV) studies using a three-electrode system. The electrochemical response presents a substantial enhancement of the area for MnO_2_-deposited electrodes compared with bare CuOHNT electrodes (Supplementary Fig. 4a). This signifies the pseudocapacitive energy storage capability to store large amounts of charge in the nanotubes of the electrodes. A similar trend of charge storage with longer charging/discharging time has been observed for the electrodes during galvanostatic charge–discharge (GCD) at a constant current density (Supplementary Fig. 4b). The high surface area of the MnO_2_-deposited CuOHNT and interconnected network of AgNW boosts the capacitance of the final electrodes to 145.15 mF cm^−2^. This is ∼10 times greater than that of a bare CuOHNT electrode. This outstanding capacitive behaviour has also been observed at constant current density charge/discharge processes (89.25 mF cm^−2^ at 1 mA cm^−2^). The tubular nanoarchitecture of the electrode materials prepared from CuOHNTs provides highly capacitive nano-layer of MnO_2_ to store a large amount of charges in the nanotubes. Moreover, the interconnected highly conductive network of AgNW created among the nanotubes facilitates the charges to transport throughout the electrode instantly. Including this synergistic effect, the highly interactive area of the electrode encounters exceptional interfacial contact at the electrode–electrolyte surface that prompts the ion transfer ability of the system and plays a key role in providing an excellent charge capacitive behaviour^27^,^28^,^29^. The hollow nanotube structure also exhibits good rate capability and nearly ideal CV curves (Supplementary Fig. 4c) at different scan rates (5–100 mV s^−1^) resulting in capacitance of 149.15 mF cm^−2^ (1193 F g^−1^) to 96.46 mF cm^−2^ (771.7 F g^−1^; Supplementary Fig. 4d; Supplementary Table 1). At different current densities (1–8 mA cm^−2^), the electrodes reveal nearly symmetrical charge/discharge profiles that represent highly reversible behaviour of the electrode materials (Supplementary Fig. 4e) and promising capacitance values of 89.25 mF cm^−2^ (714 F g^−1^) to 67.15 mF cm^−2^ (537.2 F g^−1^; Supplementary Fig. 4f). The electrochemical response of our supercapacitor is comparable or better than carbon fibre-based flexible supercapacitors^30^,^31^,^32^. A detailed comparison of the gravimetric capacitance of our electrode with other previously reported high-performing electrodes is given in Supplementary Table 4 (refs 33, 34, 35).

To make an ENHANS ribbon, we have developed a symmetric all-solid-state supercapacitor device from this CuOHNT-based electrodes. A schematic of electrode fabrication, materials characterization and electrochemical analysis of the fabricated supercapacitor is provided in Supplementary Figs 2 and 3. A concise discussion about the fabrication procedure of the supercapacitor ribbon is given in the Methods section. The cyclic voltammograms of the supercapacitor ribbon reveal quasi-rectangular redox behaviour in the pseudocapacitive transition metal oxides (Fig. 2d). Such electrochemical responses arise from the combination of faradaic reactions^36^,^37^ and the electric double-layer capacitance^23^. As discussed by Toupin *et al*.^23^, the deviation from the rectangular shape of the CV curve (Fig. 2d) is due to the polarization resistance of our cell. In addition, both slower ionic transport in solid electrolytes and the tubular nature of CuOHNTs contribute to the deviation of the voltammograms from its ideal oxidation–reduction appearance. A similar appearance is also reported by Cao *et al*.^38^ The synchronization effect of the MnO_2_-coated tubular nanostructure enables the assembled supercapacitor to deliver a capacitance of 67.78 mF cm^−2^ at 5 mV s^−1^. The areal and gravimetric capacitance obtained from the assembled supercapacitor is comparable with the previously reported charge storage devices with similar nanostructured electrodes (Supplementary Table 2)^13^,^39^,^40^,^41^,^42^,^43^,^44^,^45^,^46^,^47^. On performing different scan rates, the CV of the device represents rate capability of 68.8% and results in capacitances of 67.78 mF cm^−2^/247.51 F g^−1^ (5 mV s^−1^), 57.83 mF cm^−2^/234.36 F g^−1^ (20 mV s^−1^), 52.12 mF cm^−2^/211.22 F g^−1^ (50 mV s^−1^) and 46.64 mF cm^−2^/183.09 F g^−1^ (100 mV s^−1^) as shown in Fig. 2e. The supercapacitor is also able to withstand a very promising capacitive performance at a low current density of 1 mA cm^−2^ as well as a high current density of 8 mA cm^−2^ (Supplementary Fig. 5a). The prominent pseudocapacitive charge storage mechanism and tubular nature of our nanostructured electrode cause the charge–discharge profiles of our devices deviate from the typical straight line to a curved line (Supplementary Fig. 5a). The slow diffusion rate of de-solvated ions into the long tubular structures may affect the charging and discharging time of the electrodes^23^. Similar GCD profiles are reported for tubular electrode structures with solid electrolytes^48^. However, the capacitance values calculated from the discharge part of the GCD profiles show promising energy storage ability ranging from 48.03 mF cm^−2^ (1 mA cm^−2^) to 26.99 mF cm^−2^ (8 mA cm^−2^; Supplementary Fig. 5b; Supplementary Table 2).

The interconnected tubular nanostructures not only provide high capacitance but also exhibit negligible degradation in performance on mechanical deformation. The PVA gel electrolyte protects the device from mechanical stresses. The strong interaction between the current collector and the chemically grown CuOHNT coated with MnO_2_ results in similar electrochemical behaviour on bending at different angles as shown in Fig. 2f. The capacitive performance of the device is highly stable even after a significant amount of repeated bending (98% of initial capacitance retention at 5 mV s^−1^ after 100 bending cycles at 90°). The continuous electrochemical charge–discharge ability of the device has also been studied; CV at 50 mV s^−1^ for 10,000 cycles. The tubular nanoarchitecture of the CuOHNT buffers the volume expansion and contraction of MnO_2_ during multiple charge–discharge cycles by providing long-term structural stability of the electrodes^49^,^50^. This self-adaptive nature helps to retain a capacitance of ∼91% from the initial value even after 10,000 cycles. This indicates that the CuOHNT approach is an attractive method to develop reliable electrodes for high-performance supercapacitors. An increase of capacitance by ∼30% from the initial value is associated with the tubular nature of the nanostructure because the electrolyte ions continue to diffuse. This happens even after the initial electrochemical cycling, thereby utilizing the complete electroactive surface of the hollow nanotubes (Fig. 2g). Though the columbic efficiency from the GCD cycles of the device is not very high, the nanopores created by the long hollow tubes successfully control the volume expansion of MnO_2_, providing mechanical stability for electrodes to accomplish higher cycle life. The materials stability, interconnected nanotubular structure and ionic interaction on electrode–electrolyte surface and high cycle life makes this device comparable to other high-performing metal oxide-based long lasting energy storage systems^29^,^40^,^42^,^43^,^45^. The Nyquist plot in Fig. 2h shows that an electron impedance spectrum (EIS) after 10,000 cycles is similar to that after 50 cycles. The continuous cycling only affects the internal cell resistance resulting from long shuttling of electrolyte ions and inefficient electrical contact between the nanoarchitecture and the copper ribbon surface^51^. The angled-view SEM image of an electrode and a cross-sectional SEM image of the assembled device are given in the Supplementary Fig 6a,b, respectively. It can be seen that CuOH hollow nanotubes and AgNW network is still intact on the copper tape even after 10,000 cycles (shown in Supplementary Fig. 7).The as-prepared supercapacitor can provide a wider potential window and higher charge storage ability when they are connected in series (Supplementary Fig. 5c,d). To demonstrate the working of the supercapacitor, three devices connected in series are charged by an electrochemical workstation and used the stored charge to rotating a propeller of a toy drone (Fig. 2i; see also Supplementary Movie 1).

### Fabrication and performance of PSCs

To build PSC part for the ENHANS ribbon, we first fabricated a photo-harvesting layer of methylammonium lead iodide (CH_3_NH_3_PbI_3_) on a flexible ITO/polyethylene terephthalate (PET) substrate with a layer of hole transport layer of PEDOT:PSS on one side and PCBM on the other side as an electron transport layer. The device architecture before attaching to the shared electrode is: ITO/PEDOT:PSS/CH_3_NH_3_PbI_3_/PCBM. The post-treatment requires only low annealing temperature, which makes this device architecture a promising candidate for large-scale fabrication^52^. Solar cell fabrication and characterization details can be found in the methods part. The CH_3_NH_3_PbI_3_ perovskite layer was prepared by a chlorobenzene (CB)-assisted method to grow large perovskite crystals with grain sizes >1 μm for achieving a high performance^19^. To assess the quality and grain size of the perovskite film formed by CB-assisted growth process with respect to that of the control sample (non-assisted growth), we inspected top-view SEM image (Supplementary Fig. 8a,b) of the CB-assisted growth film and control sample. The film obtained by CB-assisted method exhibits considerably large crystal grains (∼1 μm) and almost full coverage on the substrate without any pinholes, while the control sample shows small crystal grains (300 nm) with pinholes. After coating PCBM on top of a perovskite layer, copper ribbon with the conductive adhesive side of the supercapacitor was pressed on top of the PCBM layer to complete the ENHANS ribbon. It is to be noted that the adhesive on the reverse side of the copper ribbon is highly conductive and the assembled ribbon is mechanically stable as a single integrated device as evidenced by the bending tests of the device discussed in the following section. A comparison of the solar cell *I*–*V* curves for a copper ribbon electrode and a deposited copper film electrode is shown in supplementary Fig. 9. This comparison demonstrates that the conductive adhesive on the copper tape does not affect the performance of the solar cell considerably.

**Figure 3:**
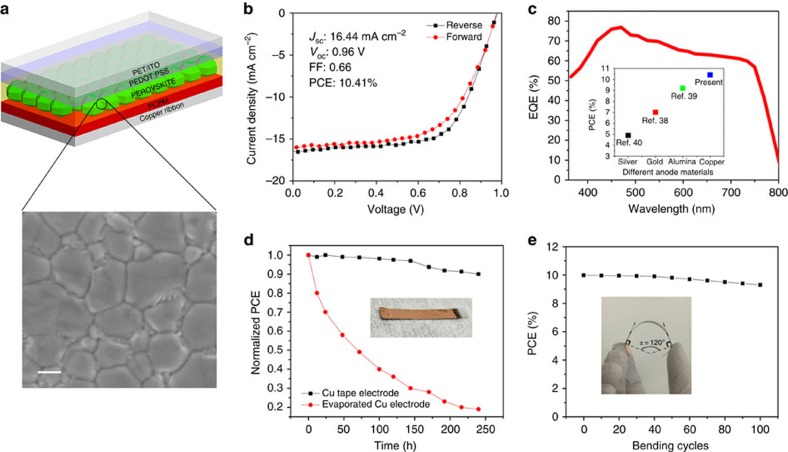
Device performance of perovskite solar cell with respective device structures. (**a**) The schematic of a flexible perovskite solar cell with Cu tape anode. Zoomed-in region of the perovskite layer represents SEM image of perovskite grain crystal with 1 μm crystal grain size. Scale bar, 500 nm. (**b**) *I–V* curve of the best device under 1 sun conditions (100 mW cm^−2^, AM 1.5 G). (**c**) EQE versus wavelength for the device; inset shows a comparison of the present PCE with other reported inverted flexible PSC using different thermal evaporated negative electrodes. (**d**) Normalized PCE of PSC made with Cu tape electrode and thermally evaporated Cu electrodes after being stored in air for 240 h. (**e**) PCE stability based on the bending cycles for PSCs with a 120° angle; inset shows the photograph of the bending process and angle determination.

An independent copper ribbon PSC provides a short-circuit current density (*J*_sc_) of 16.44 mA cm^−2^, open-circuit voltage (*V*_oc_) of 0.96 V, fill factor of 0.66 and a power conversion efficiency of 10.41% for reverse scan as shown in Fig. 3b. The *J*_sc_ matched well with the current density of 16.6 mA cm^−2^ calculated from external quantum efficiency (EQE) measurement (Fig. 3c). The inset of Fig. 3c shows our PCE using CB-assisted growth and a comparison with other reported inverted flexible PSC using different thermal evaporated negative electrodes: gold (Au) and aluminum (Al). Carmona *et al*.^53^ reported inverted flexible PSCs comprised of Ag/PEDOT:PSS/PolyTPD/CH_3_NH_3_PbI_3_/PCBM/Au exhibiting an efficiency of 7%. In this work, an additional electron block layer-PolyTPD has been inserted in between PEDOT:PSS and perovskite layer. You *et al*.^54^ reported a PCE of 9.2% for a mixed halide (CH_3_NH_3_PbI_3−*x*_Cl_*x*_) solar cell composed of ITO/PEDOT:PSS/MAPbI_3−*x*_Cl_*x*_/PCBM/Al. Compared with the above thermal evaporation processes for making Au- or Al-negative electrode, our copper ribbon electrode is less expensive and require shorter processing time. By using flexible copper ribbon as an anode, a high PCE of 10.41% was achieved along with a much improved environmental stability that is very important for commercial applications. The copper ribbon as the top electrode plays an important role for enhancing the stability of PSCs because it effectively blocks the permeation of moisture and oxygen into the perovskite layer and our device delivers one of the best performances of sandwich-type PSC. As shown in Fig. 3d, the laminated PSCs have superior lifetimes than devices with 100 nm thermally evaporated Cu electrodes. After exposing it to air for 10 days, the normalized PCE of devices with Cu tape electrodes still shows 90% of their initial efficiencies, while devices with evaporated Cu electrode degrades significantly from 100 to 20%. It is to be noted that further improvement in stability can be achieved by sealing the edges of our ribbons that is not presently sealed and protected. Also, our current results represent one of the best performances with respect to the environmental stability of flexible PSCs^55^,^56^. Another advantage of this design is that the device exhibits good mechanical flexibility. The PCE of the flexible PSCs is measured after being bent repeatedly at 120° angle. The PCE shows minimal deterioration even after bending 100 times, retaining 90% of its initial PCE value (Fig. 3e) due to high flexibility and adhesive property of copper tape/ribbon. The X-ray diffraction measurement was used to investigate the crystallinity of the perovskite film (Supplementary Fig. 8c). Two peaks can be identified at 14.100 and 28.470, which are assigned to 110 and 220 diffraction peaks for the CH_3_NH_3_PbI_3_ material with a tetragonal crystal structure^57^. The ultraviolet–visible absorption measurement was performed (Supplementary Fig. 8d) to demonstrate the promising absorption property of perovskite layer.

### Performance and weaving of the ENHANS ribbons

**Figure 4:**
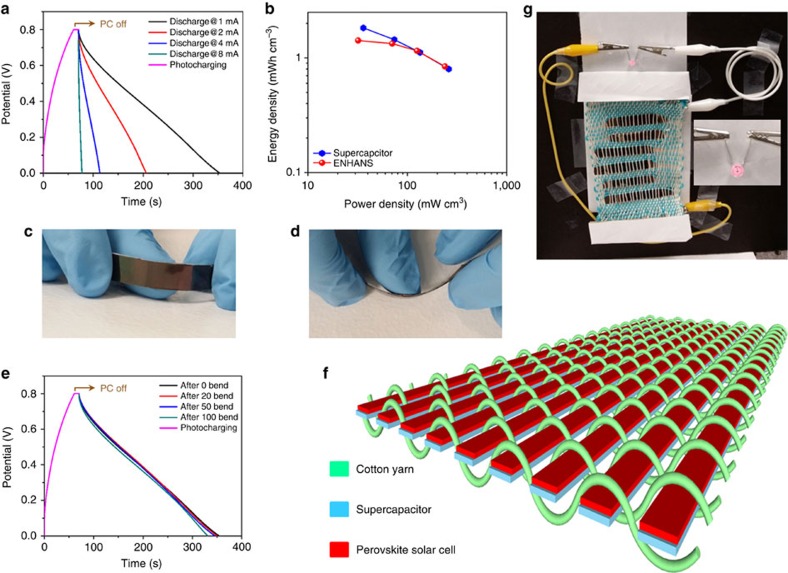
Simultaneous energy harvesting and storage tests. (**a**) Charge–discharge profile of the ENHANS ribbon. The solar side of ENHANS ribbon has been charged with the solar simulator for 1 min and discharged with electrochemical workstation at different current densities after 10 s photocharging (PC) off; (**b**) Ragone plots of an independent supercapacitor and an ENHANS ribbon to compare the energy density and power density at different charging–discharging rate (1, 2, 4 and 8 mA cm^−2^ current density); (**c**,**d**) photograph of the ENHANS ribbon being bent at different angles; (**e**) charge–discharge profile of the ENHANS ribbon after a different bending cycles (the ENHANS ribbon has been photocharged for 1 min, and then removed from light 10 s before discharge); (**f**) schematic illustration of the ENHANS ribbon after weaving with the cotton yarn to make a portable lightweight cloth and (**g**) ENHANS ribbons weaved with cotton thread to demonstrate the working of the lightweight fabric. The photograph shows the charge deliverability of weaved matrix as a result of one minute photocharging.

After testing the performance of the independent devices, the synergistic effect of the integrated device is tested and confirmed. In the integrated ENHANS ribbon of 1cm^2^ area, the solar cell part is able to generate ∼4 mA current with photocharging process. The charges generated at the PSC are directly delivered to charge the supercapacitor through the shared electrode according to our proposed mechanism in Fig. 1b. The shared copper electrode provides high conductivity that enables similar energy efficiency for the integrated solar ribbon as well as the standalone PSC. This can be clearly seen from the *I*–*V* curve given in Supplementary Fig. 10. The charge transfer from solar cell to supercapacitor can fully charge supercapacitor to a potential of ∼0.8 V when the solar cell is illuminated with 1 sun. In the integrated ENHANS filament, the solar cell component is able to generate a maximum current of ∼10 mA by photocharging with a solar simulator (1 sun) within a minute. The energy harvested by the PSC is directly delivered to charge the supercapacitor through the shared electrode. The voltage increases considerably in the first few seconds and reaches a constant value of ∼0.8 V. Therefore, as photocharging take place, the photogenerated current increases until the voltage of the supercapacitor reaches the *V*_OC_ of the solar cell. The photocurrent from the solar cell decreases once it reaches its maximum voltage. In addition, we have photocharged the ribbon for 1 min and discharged with electrochemical workstation at various current densities (1–8 mA cm^−2^) after waiting for 10 s since the seizure of photocharging (Fig. 4a). This indicates that the harvested energy by the solar cell stored in the supercapacitor part of the device is available when necessary. By using the discharge time in Fig. 4a, we have calculated and plotted the energy density as 1.82 mWh cm^−3^ and power density 36.19 mW cm^−3^ at 1 mA cm^−2^ current density of the ENHANS ribbon and compared its performances with the independent supercapacitor Fig. 4b; Supplementary Tables 2 and 3. The Ragone plot in Fig. 4b shows that the performance of the supercapacitor in the ENHANS ribbon is similar to a solo supercapacitor. By photocharging at 1 sun, the ENHANS provides an energy density of 1.15 mWh cm^−3^ and a power density of 125.25 mW cm^−3^, which is comparable with the previously reported independent supercapacitor and other similar devices^29^,^40^,^41^,^42^,^43^,^47^,^58^,^59^. The energy and power density of the ENHANS ribbons are slightly inferior at lower current density discharge rate (1 and 2 mA cm^−2^) compared with a solo supercapacitor when it is charged/discharged with electrochemical work station at similar current density (Supplementary Table 1). When photocharged with 1 sun, the amount of charge stored in the ENHANS is lower compared with the solo supercapacitor charged with the electrochemical workstation at 1 or 2 mA cm^−2^. In addition, the ENHANS ribbons discharge quicker compared with the single SSC and experience lower energy as well as power densities. However, at a higher current density discharge rate, the energy and power density of the ENHANS ribbon is similar to the supercapacitor itself as it is photocharged with 1 sun (Fig. 4b).

It is found that ∼67% of the energy harvested by the solar cell is stored in the ENHANS^60^. There are a few pathways that self-discharge could take place in the charged ENHANS ribbon. The self-discharge of the device could be due to a continuous faradaic charge transfer which leads to a leakage current passing across the electrochemical double layer. It could also occur due to the relaxation of the potential from the supercapacitor to the other regions of the device that are at a lower potential. Diffusion-controlled depolarization could also happen if redox impurities are present in the electrode or electrolyte. To measure the leakage current, we applied a d.c. voltage to the supercapacitor and measured the current required to maintain the voltage^61^. The monitored supercapacitor showed a leakage current of 5 μA after 90 h as given in the Supplementary Fig. 11.

With our low temperature, simple fabrication method, the lightweight ENHANS ribbon exhibits high flexibility. To demonstrate this, we have carried out a bending test on our ribbons by bending the device at 90° for a certain number of times (20, 50 and 100 at 90° angle; Fig. 4c,d) and subjected the device to photocharging and discharging (discharged at 1 mA cm^−2^ current density). The flexible nature of the electrodes provides the ENHANS ribbon the ability to withstand external mechanical deformation. In Fig. 4e, the discharge curve of the device after a certain number of bending cycles is almost identical to the electrochemical response of a device without bending. Since we used wet processing technique for depositing all layers except the electrodes, a roll-to-roll process could be a potential manufacturing technique for large-scale manufacturing of ENHANS ribbons, similar to the process discussed for Li-ion batteries^62^,^63^.

The interlacement of ribbons with ENHANS ribbons into a textile provides added mechanical strength. This type of woven structure enables the introduction of various types of ribbons to meet the requirements of a given application in which the device is being used. A plurality of ribbons can be woven into a single fabric structure. Figure 4f is a schematic showing the ENHANS ribbons woven into a textile form with supporting cotton yarns (green line). To demonstrate the working of ENHANS ribbons as a fabric for simultaneous energy generation and storage, the matrix was exposed to a solar simulator (1 sun, AM 1.5 G) to photocharge for 1 min (S1 open and S2 closed) and used the stored charge to light an light-emitting diode after ceasing the photocharging (Fig. 4g; see also Supplementary Movie 2). The discharge profile of the weaved ENHANS ribbons (shown in Fig. 4g) photocharged using 1 sun is provided in Supplementary Fig. 5e. However, when S1 and S2 in Fig. 1 are closed simultaneously, the energy from the solar cell is released directly to power the load (light-emitting diode) instead of storing energy in the supercapacitor, as demonstrated in Supplementary Movie 3.

## Discussion

We have developed a single ribbon that simultaneously harvests and stores energy. The energy generated on one side of the ribbon can be stored on the other side through a shared electrode. The PSC developed provides a high-energy conversion efficiency of >10% with high environmental stability. A simple wet processing technique is used to fabricate CuOHNT on the supercapacitor electrodes that considerably enhance the storage capability of the ENHANS ribbons. The high flexibility and all-solid-state device architecture of the ENHANS ribbons provides the ability to weave them into a textile form which may find numerous applications including wearable electronics.

## Methods

### Supercapacitor component

Cu(OH)_2_ nanotube (CuOHNT) arrays are grown directly on a copper-tape (copper ribbon) substrate via a simple one-step reaction reported elsewhere^24^,^25^,^26^. The copper tape with adhesive side masked is introduced into a chemical bath consisting of 3 g of NaOH, 0.684 g of (NH_4_)_2_S_2_O_8_ and 30 ml of de-ionized (DI) water. After ∼20 min submersion, the colour of the copper samples began to turn into a light shade of blue, indicating the growth of Cu(OH)_2_ nanotubes. To improve charge transport between the tubes, Ag nanowires (Blue Nano) dispersed in isopropyl alcohol at a concentration of 1 mg ml^−1^ are drop cast (6 μl) onto the Cu(OH)_2_ electrodes and left to dry for 6 h at 45 °C in an oven^64^. Addition of AgNW between CuOHNT provides an interconnected conductive network among the nanotube architecture (Supplementary Fig. 3c). To make the charge transport more efficient, we deposited a thin layer of copper or gold–palladium (AuPd) by sputter coating (Supplementary Fig. 3d) at a constant current of 40 mA for 6 min on top of the CuOHNT and AgNW. AuPd coating provides a better conductivity compared with Cu (refs 39, 65). This highly conductive matrix constitutes a favourable surface for MnO_2_ electrodeposition^39^ on/into the tubular nanoarchitecture (Supplementary Fig. 3e). MnO_2_ is deposited for 12 min onto the nanostructures via an anodic electrodeposition method at a constant current density of 0.5 mA cm^−2^. The electrolyte for electrodeposition was prepared by dissolving 0.01 M manganese acetate and 0.02 M ammonium acetate into a solvent containing 10 v/v % of dimethyl sulfoxide and 90 v/v % of DI water. Supplementary Fig. 3f,g shows the TEM images of MnO_2_-deposited CuOHNT as well as the layer thickness of the deposited material on the final electrodes. The loading of the MnO_2_ in a single electrode is ∼0.2 mg. The electron loss spectroscopy (ELS) in TEM is explored to monitor the MnO_2_ morphology on the nanotubes surface as shown in Supplementary Fig. 3h. The X-ray photoelectron spectroscopy survey spectrum in Supplementary Fig. 3i reveals the presence of Au (84 eV), Pd (336 eV), Ag (378 eV), Cu (934 eV) and Mn (643 eV) in the final composition of the MnO_2_-deposited nanotubes^65^,^66^. This indicates that the deposition of target materials such as MnO_2_ provides a smooth nanostructure on the CuOHNT and two peaks of MnO_2_ represent the Mn^4+^ oxidation state to experience excellent electrochemical response. Moreover, the tubular nature of the high aspect ratio nanostructures on the electrode surface provides highly enhanced surface area and high ion mobility at the electrode–electrolyte interface in contact with electrolyte ions^28^,^67^,^68^.

The finalized supercapacitor device is then fabricated by wetting the electrode surface with 1 M KOH/PVA gel electrolyte and allowing it to partially dry. Two symmetric electrodes are then pressed together with a pressure of 1 Mpa for 1 h. All electrochemical performances were recorded after cycling the electrodes 20 times.

### PSC component

The patterned ITO/PET substrate in the same shape (ribbon form) and size as the Cu tape is subsequently cleaned in an ultra-sonicator with DI water, acetone and isopropanol for 15 min each. On the cleaned ITO substrate, a thin layer of PEDOT:PSS (Clevios P VP AI 4083) is deposited by spin coating and annealed at 120 °C for 30 min. Subsequently, the substrate is transferred into a glove box for the deposition of perovskite absorber and PCBM layers. Perovskite precursor solution is prepared by mixing CH_3_NH_3_I and PbAc_2_ in anhydrous *N*,*N*-dimethylformamide at a 3:1 molar ratio with a final concentration of ∼40 wt%. The filtered perovskite precursor is spin-casted at 3,000 r.p.m. for 45 s and after 6 s anhydrous CB (150 μl) is quickly dropped onto the center of the sample. The role of CB is to rapidly reduce the solubility of CH_3_NH_3_PbI_3_ in the mixed dimethylformamide (solvent for perovskite precursor) and CB. This procedure promotes fast nucleation and growth of the crystal. The colour of the film is turned from colourless to light brown instantly. The resulting film was annealed at 100 °C for 10 min to achieve large grain size for perovskite films. The thickness of the CH_3_NH_3_PbI_3_ layer is ∼350 nm. PCBM solution is then spin-casted onto the CH_3_NH_3_PbI_3_ layer at 1,000 r.p.m. for 45 s. For solar cell *I*–*V* measurement, copper ribbon is used as anode for performing *I*–*V* and EQE of the device.

### Fabrication of the ENHANS

After assembling this all-solid-state supercapacitor in symmetric configuration, the mask on the reverse side was removed from the copper tape to expose its conducting adhesive. This is followed by pressing the PCBM layer of perovskite solar part fabricated on top of flexible ITO/PET onto the adhesive side of the copper tape. This integrates the solar part on top of the supercapacitor part to share the copper ribbon as a single electrode (negative electrode). To transfer the harvested charge from solar cell to the supercapacitor for storage, one end of the top copper electrode of supercapacitor is directly connected to the positive electrode (PET/ITO) of solar cell through a switch (Fig. 1e).

### Weaving of the energy-smart textile

Weaving is a versatile well-established technique to interlace continuous longitudinal (warp) filaments with horizontal filaments. The horizontal filaments can be either continuous; that is, a single filament that is repeatedly inserted across the width of the fabric or segments that can be interlaced as shown in Supplementary Fig. 12. The technology lends itself to mass production as well as development of small scale prototypes. The flexible ENHANS device is introduced as horizontal (weft) filament that is interlaced by the warp filament to provide mechanical stability and structural integrity to the fabric. Further details of weaving a fabric using ENHANS ribbons are discussed in the Supplementary Methods. This enables the ENHANS device to protrude from the edge of the fabric and can be interconnected to complete the circuit. A subset of filaments may be used to provide mechanical strength, form and packaging to the electrically active filament component. Furthermore, the fabric developed can be easily folded that is essential for portable energy applications (Supplementary Fig. 12d). The textile supporting structure allows the device to be gently compressed and folded while ensuring that contacts critical to the completeness of the circuit are not damaged.

### Device characterization

For the evaluation of the PSCs performance, a solar simulator with a 300 W Xenon lamp and AM 1.5 G global filter were used. The light intensity, 100 mWcm^2^, was calibrated using a silicon photovoltaic reference cell (Newport, 91150 V). Current–voltage characteristics were measured with a Keithley 2635A source measurement unit. Devices were masked with a black metal aperture to define an active area of 0.075 cm^2^. The EQE spectra were measured using a Newport model QE-PV-SI coupled with a lock-in amplifier and a calibrated silicon photodetector. Histogram of PCEs measured for 48 solar cells prepared by CB-assisted growth technique is given in the Supplementary Fig. 13. Surface morphologies were characterized by means of SEM (ZEISS Ultra 55) and TEM (TECNAI F30) equipped with an energy-dispersive X-ray spectroscopy. To investigate the chemical compositions of sample surface, X-ray photoelectron spectroscopy (PHI 5400) was performed. To compare the electrochemical performance of different materials, a three-electrode system consisting of a working electrode (based on the materials prepared), a platinum counter electrode and a saturated calomel electrode as reference electrode were used. CV and GCD measurements were performed using this three-electrode configuration in 1 M KOH solution using an electrochemical workstation (Bio-Logic, SP-150). The characterization experiments of the devices using solid electrolyte were conducted by a two-electrode system in air at a voltage range of 0–0.8 V. Electrochemical impedance spectroscopy (EIS) measurements were performed by applying an a.c. voltage with 5 mV amplitude in a frequency range from 10 mHz to 100 kHz. All calculations including specific capacitance as well as energy density and power densities are discussed in the Supplementary Information. For testing environmental and operational stability^69^,^70^, the solar cell was kept under an atmosphere with ∼50% humidity for 240 h. The PCE was measured once every 30 min under continuous illumination for the first 5 h. After that, the device was left under a room light in the open atmosphere and the PCE was monitored once every 24 h for 10 days. The plot of PCE versus time is shown in Supplementary Fig. 14.

### Data availability

The data that support the findings of this study are available from the corresponding author on request.

## Additional information

**How to cite this article:** Li, C. *et al*. Wearable energy-smart ribbons for synchronous energy harvest and storage. *Nat. Commun.* **7,** 13319 doi: 10.1038/ncomms13319 (2016).

**Publisher's note:** Springer Nature remains neutral with regard to jurisdictional claims in published maps and institutional affiliations.

## Supplementary Material

Supplementary Information Supplementary Figures 1-14, Supplementary Tables 1-4, Supplementary Methods and Supplementary References

Supplementary Movie 1 Three devices connected in series are charged by an electrochemical workstation and used the stored charge to rotate the propeller of a toy drone.

Supplementary Movie 2 To demonstrate the working of ENHANS ribbons as a fabric for simultaneous energy generation and storage, the smart textile is exposed to a solar simulator to photo-charge for 1 min without connecting to any load (LED) and used the stored charge to light an LED after ceasing the photo-charging.

Supplementary Movie 3 To demonstrate that the energy from the solar cell can be directly released to power an LED without storing in the supercapacitor, the smart textile is directly connected to the LED and blocked the light intermittently.
