# Supplementary material for: Wearable energy-smart ribbons for synchronous energy harvest and storage
Source: Nat Commun. 2016 Nov 11;7:13319. doi: 10.1038/ncomms13319 (PMC5114596; doi:10.1038/ncomms13319)
Supplement: Supplementary Information — Supplementary Figures 1-14, Supplementary Tables 1-4, Supplementary Methods and Supplementary References [file ncomms13319-s1.pdf]

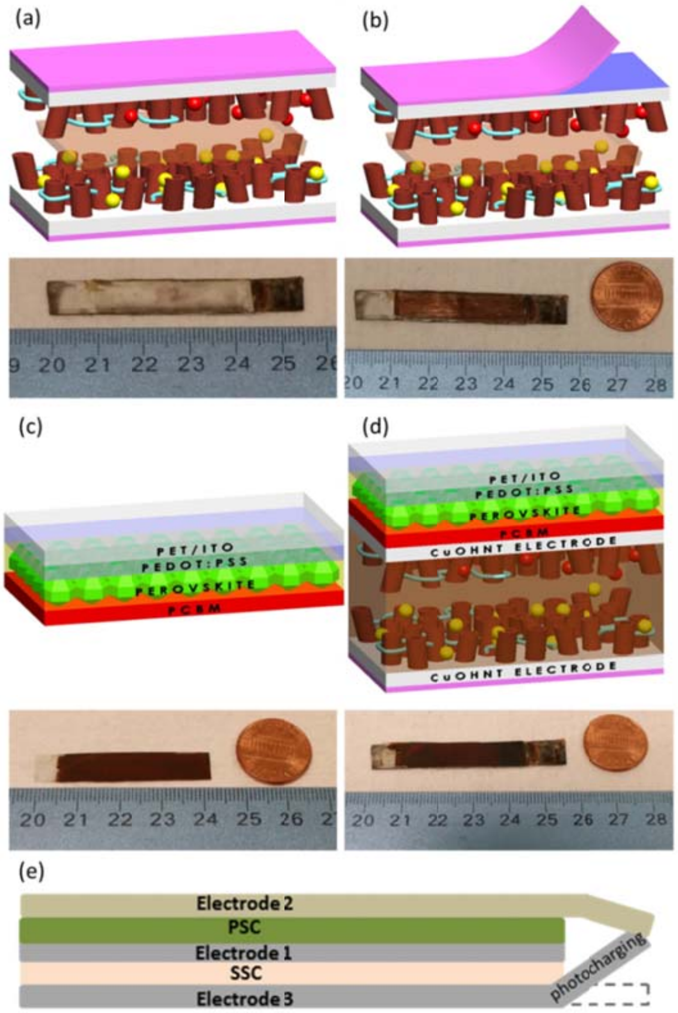

Supplementary Figure 1. Procedure for fabricating ENHANS ribbon. (a) All solid state symmetric supercapacitor, (b) supercapacitor after peeling off the paper cover from one of the copper tape electrodes side to expose the sticky nature of the charge storage device, (c) multilayer energy harvesting solar active perovskite materials partly coated on transparent PET-ITO and (d) integrated the solar active part on top of the supercapacitor sticky part side to share the copper tape as a single electrode, (e) one end of the positive electrode (electrode 2; top electrode) of the solar cell is directly connected to the cathode (electrode 3; bottom electrode) of the supercapacitor through a switch.

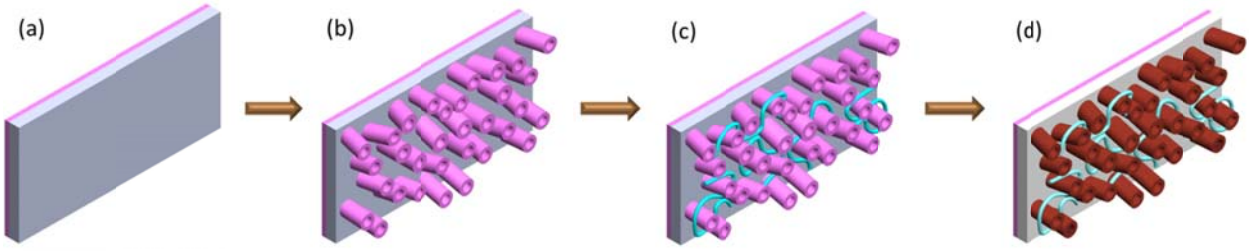

Supplementary Figure 2. Schematic illustration of the MnO_2 deposited $Cu(OH)_2$ nanotubular architecture. (a) Commercial copper tape, (b) $Cu(OH)_2$ nanotubes grown on Cu tape at room temperature by a solution method, (c) $Cu(OH)_2$ nanotubes on Cu tape after deposition of Ag-nanowires and (d) final electrode of Cu-tape obtained after MnO_2 electro-deposition on tubular $Cu(OH)_2$ structure.

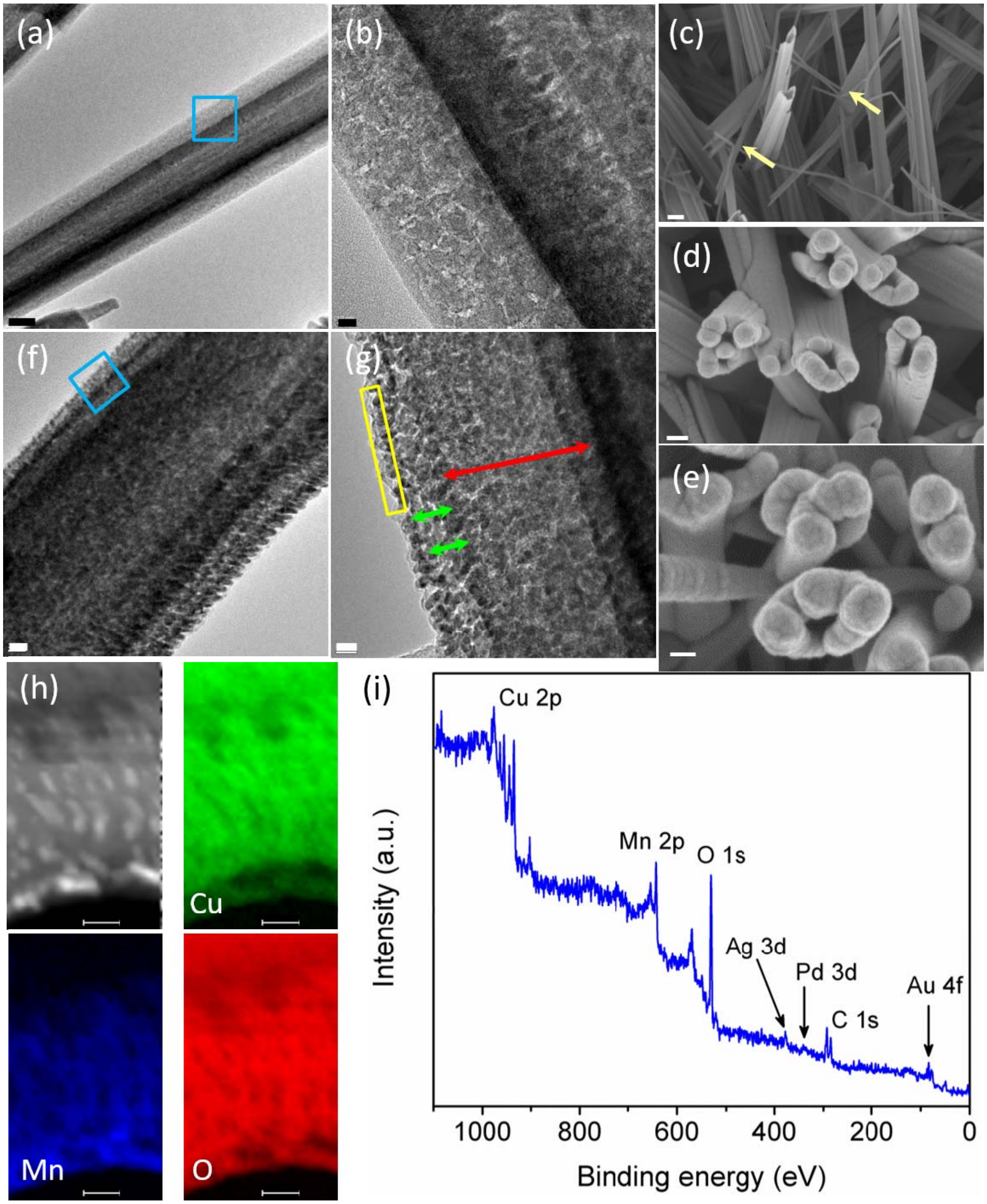

Supplementary Figure 3. Structural and compositional analysis of nanofabricated MnO_2 deposited interconnected CuOHNT. (a) Low (scale: 1 µm) and (b) high (scale: 10 nm) resolution TEM images of CuOHNT reveals the internal hollow architecture of as prepared nanotubes, (c) high resolution SEM images after AgNW (marked with arrows) deposition (scale: 200 nm), (d) high resolution FESEM of nanotubes after AuPd coating

(scale: 200 nm), (e) high resolution SEM images of MnO_2 deposited nanotubes (scale: 100 nm), (f) low (scale: 20nm) and (g) high resolution (scale: 10nm) TEM images of MnO_2 deposited nanotubes (red arrow shows the thickness of the CuOHNT walls, green arrow represents the AuPd layer on nanotube as well as yellow box is used to indicate the electro-deposited MnO_2 layer on the nanotubes. ELS mapping image of a selected area of nanotubes (h) to reveal the elemental (Cu, Mn and O) distribution on the nanotubes surface (scale: 10 nm) and (i) XPS survey analysis of the electrode material.

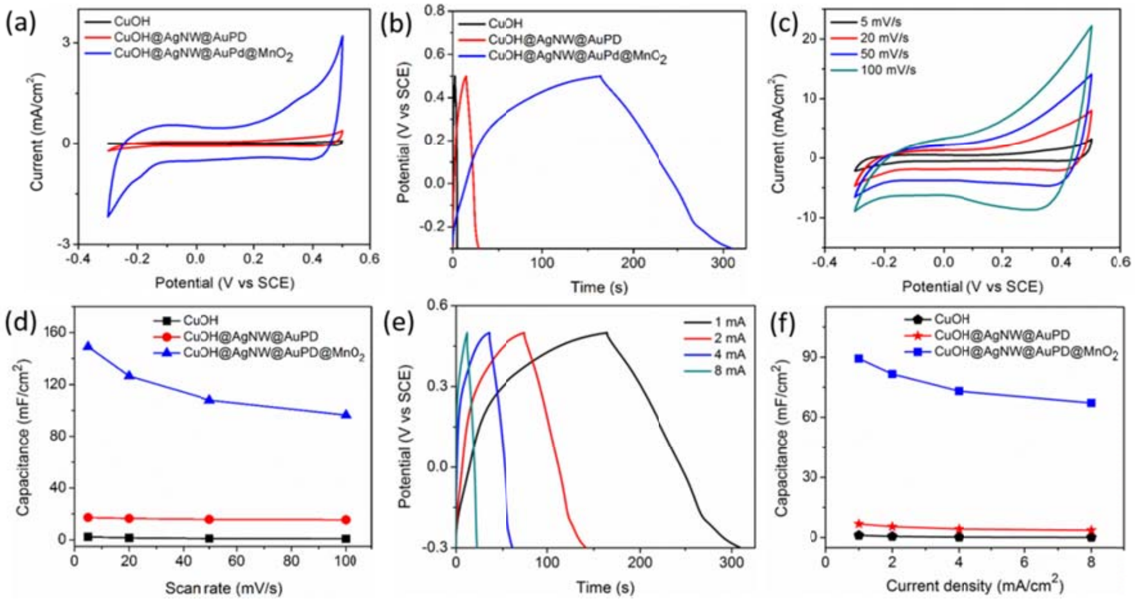

Supplementary Figure 4. Electrochemical analysis of the single electrode in a three electrode system. (a) CV study of the electrodes as different materials are deposited on $Cu(OH)_2$ nanotubes, (b) GCD study as various layers are deposited on the electrode, (c) CV of the electrode after 12 min MnO_2 electrode deposition at different scan rates, (d) specific capacitance calculated from CV study, (e) GCD of the electrode after 12 min MnO_2 electrode deposition at different scan rates and (f) specific capacitance calculated from CV study.

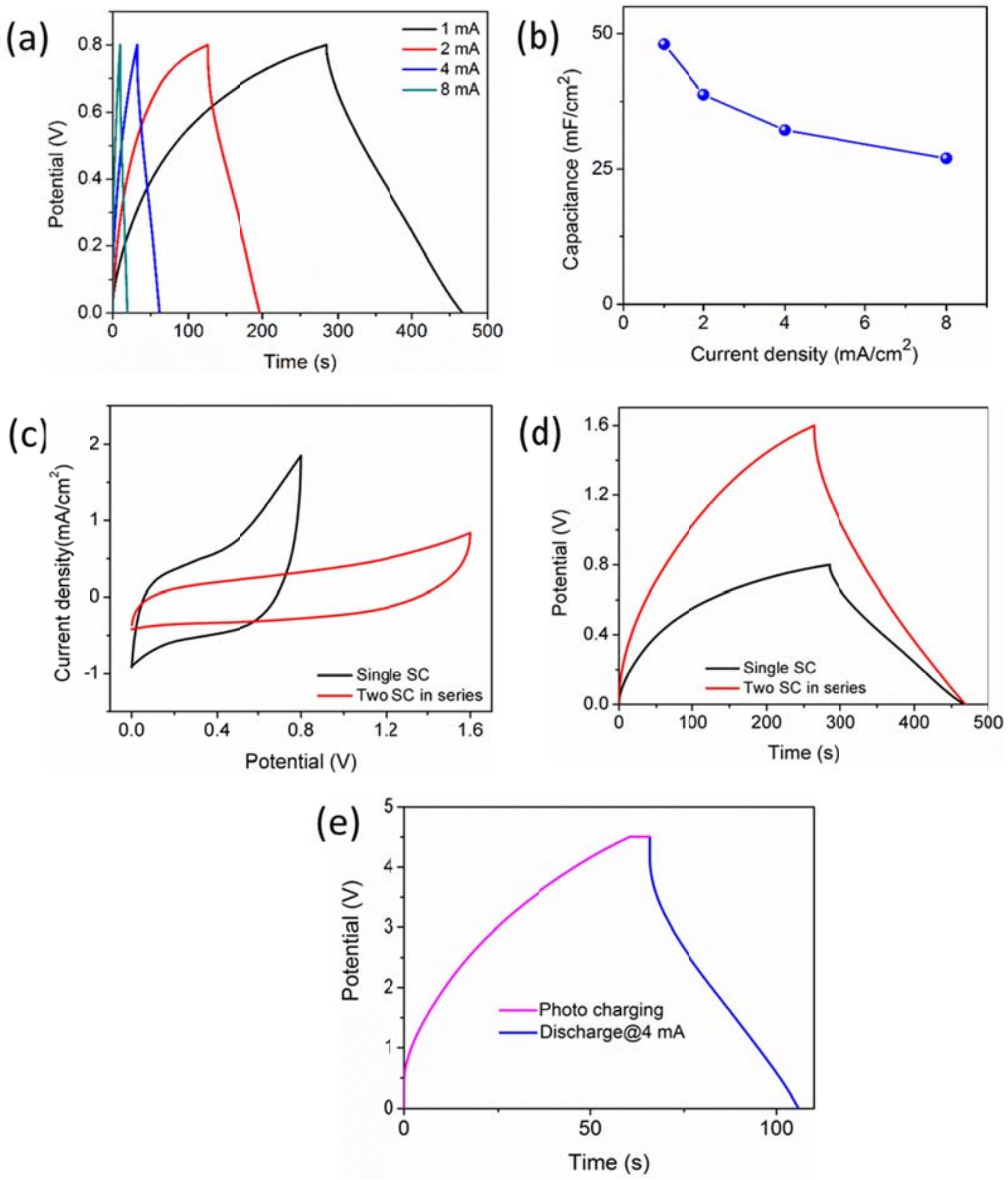

Supplementary Figure 5. Electrochemical analysis of the symmetric supercapacitor. (a) Charge-discharge study of the device at different current densities, (b) cell capacitance calculated from the GCD, (c) CV of a single supercapacitor compared to the CV of two supercapacitor in series at 5 mV s^{-1} and (d) GCD of a single supercapacitor compared to the GCD of two supercapacitors connected in series at 1 mA cm-^{2} current density, (e) the characterization of charge-discharge profile of the ENHANS ribbons weaved with cotton threads to demonstrate the working of the light weight fabric.

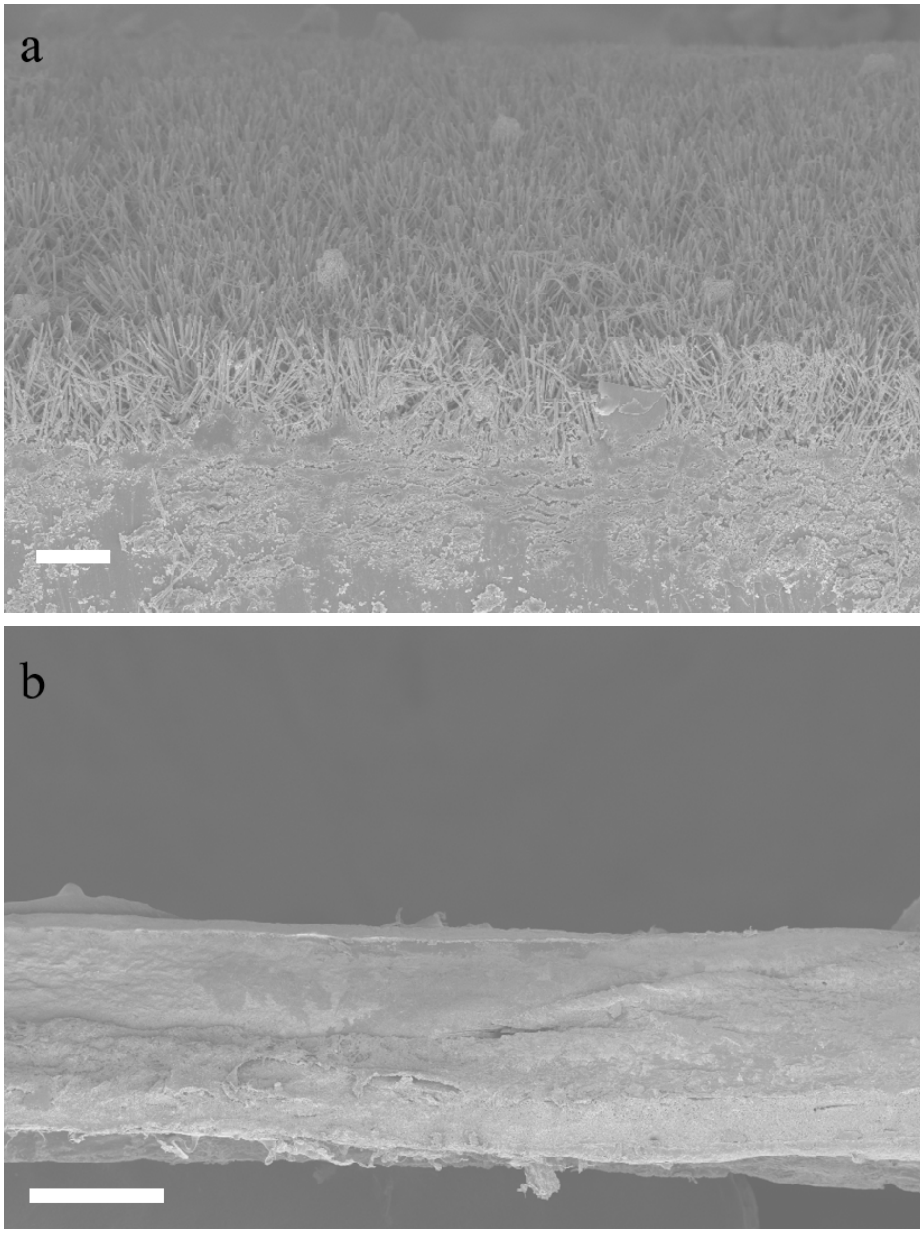

Supplementary Figure 6. (a) The angled view SEM image of an electrode (Scale: 10 μm) and (b) a cross sectional SEM image of the assembled device (Scale: 200 μm).

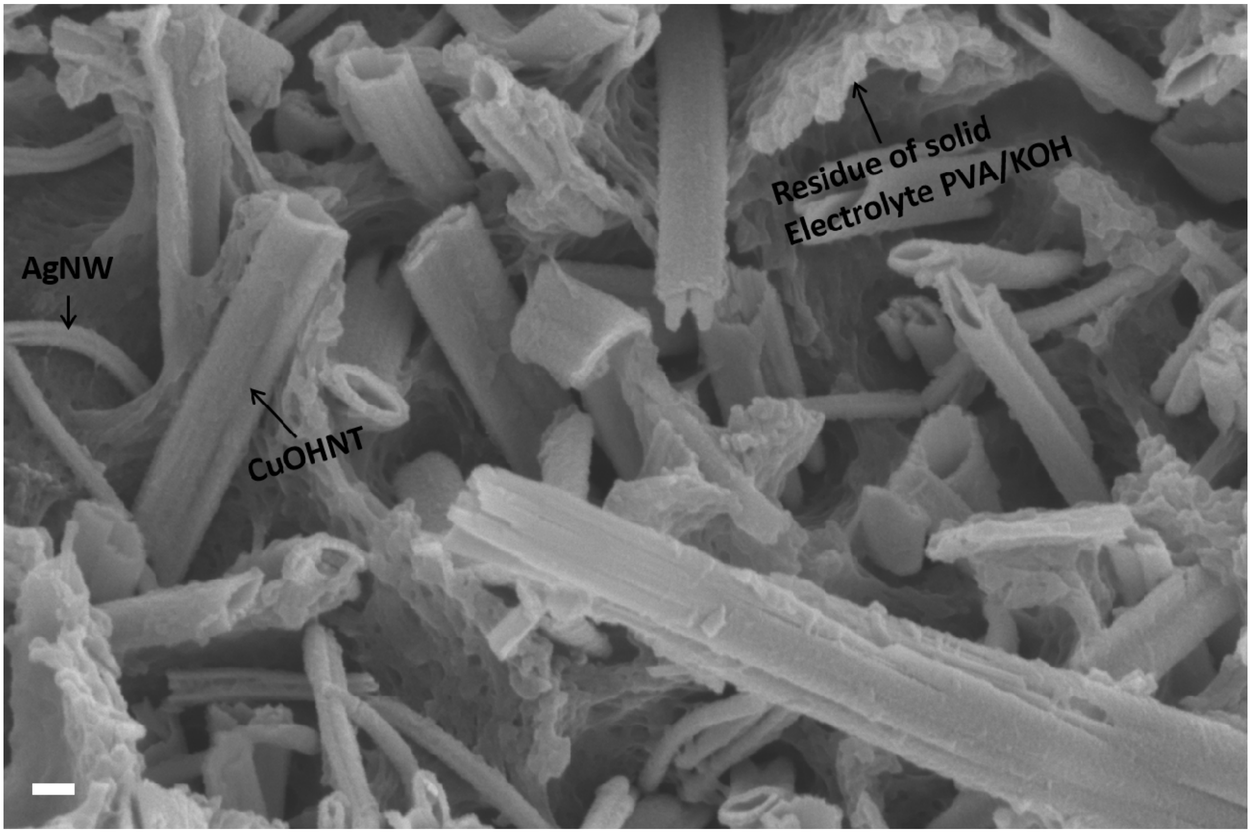

Supplementary Figure 7. High resolution SEM image of the supercapacitor after 10,000 cycles (Scale: 200 nm).

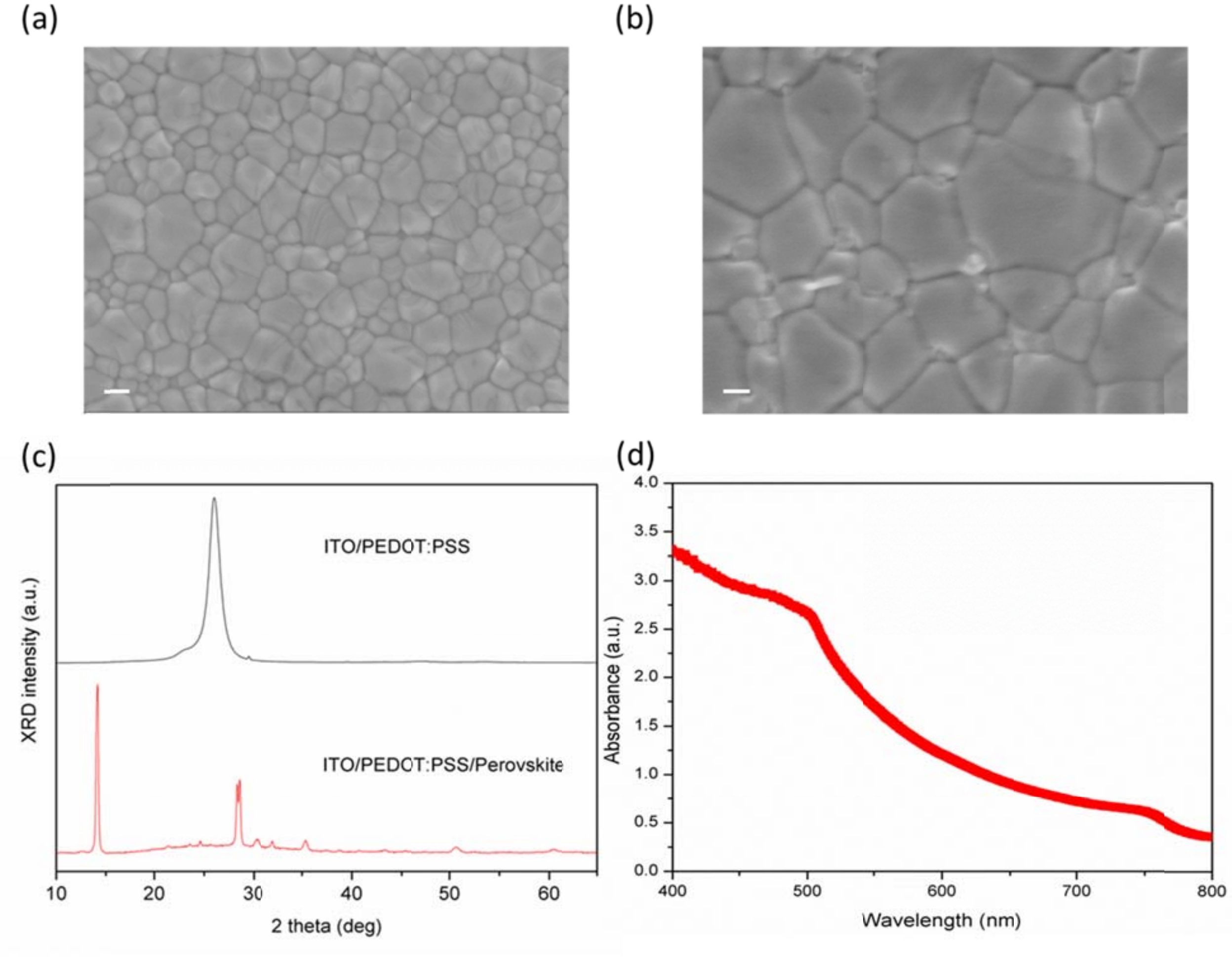

Supplementary Figure 8. Material characterization of $CH_3NH_3PbI_3$ perovskite films. (a) SEM image of a control sample grown perovskite film by traditional method, (b) SEM image of sample grown perovskite film by CB-assisted growth method (Scale: 200 nm for both). (c) XRD pattern of the perovskite crystals grown on PEDOT:PSS/ITO (red line), with blank sample of the PEDOT:PSS coated ITO substrate for comparison (black line). (d) UV-vis absorbance of $CH_3NH_3PbI_3$ perovskite films on PET substrate.

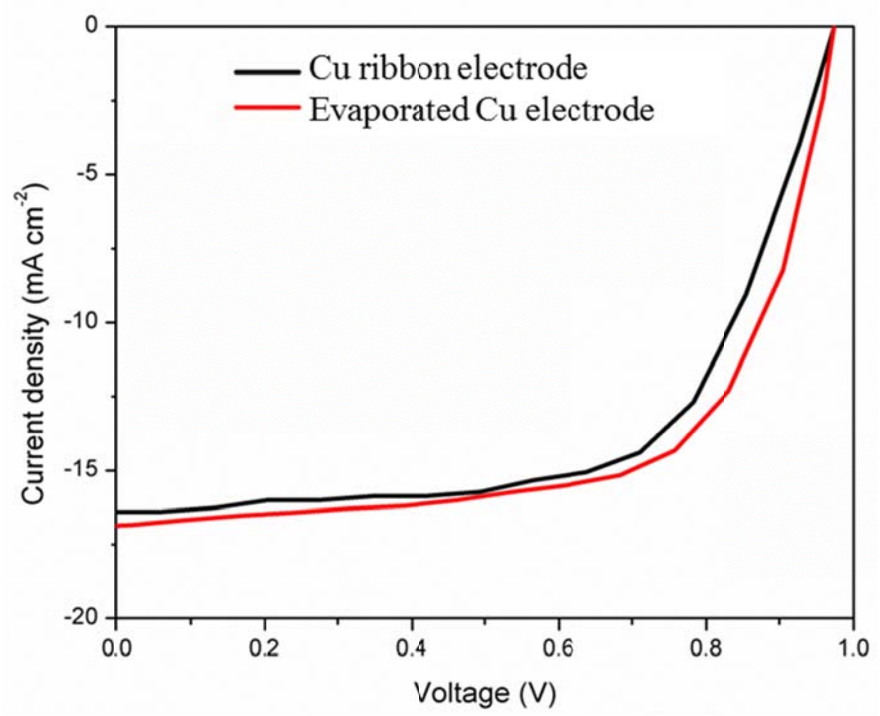

Supplementary Figure 9. A comparison of the solar cell I-V curves for copper ribbon electrode and deposited copper film electrode.

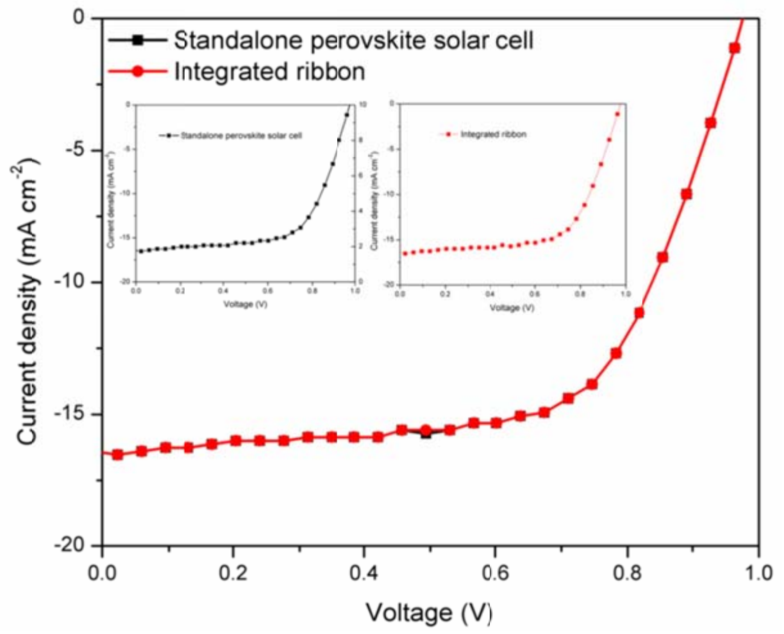

Supplementary Figure 10. The energy efficiency of the standalone perovskite solar cell and integrated ribbon.

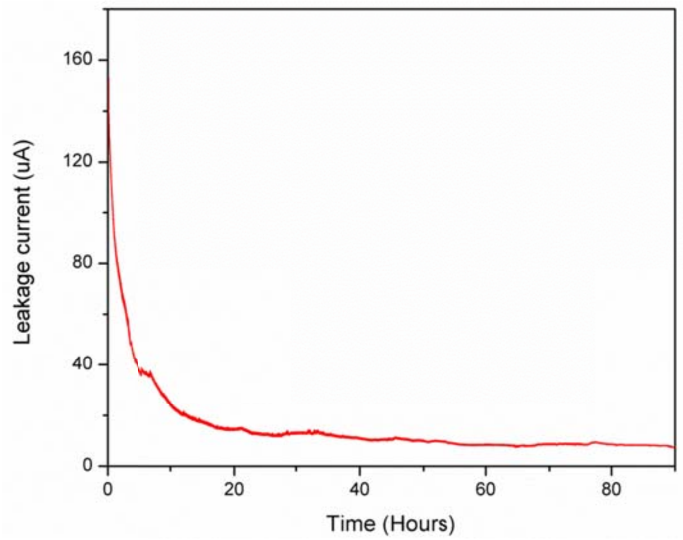

Supplementary Figure 11. The leakage current vs time in the supercapacitors.

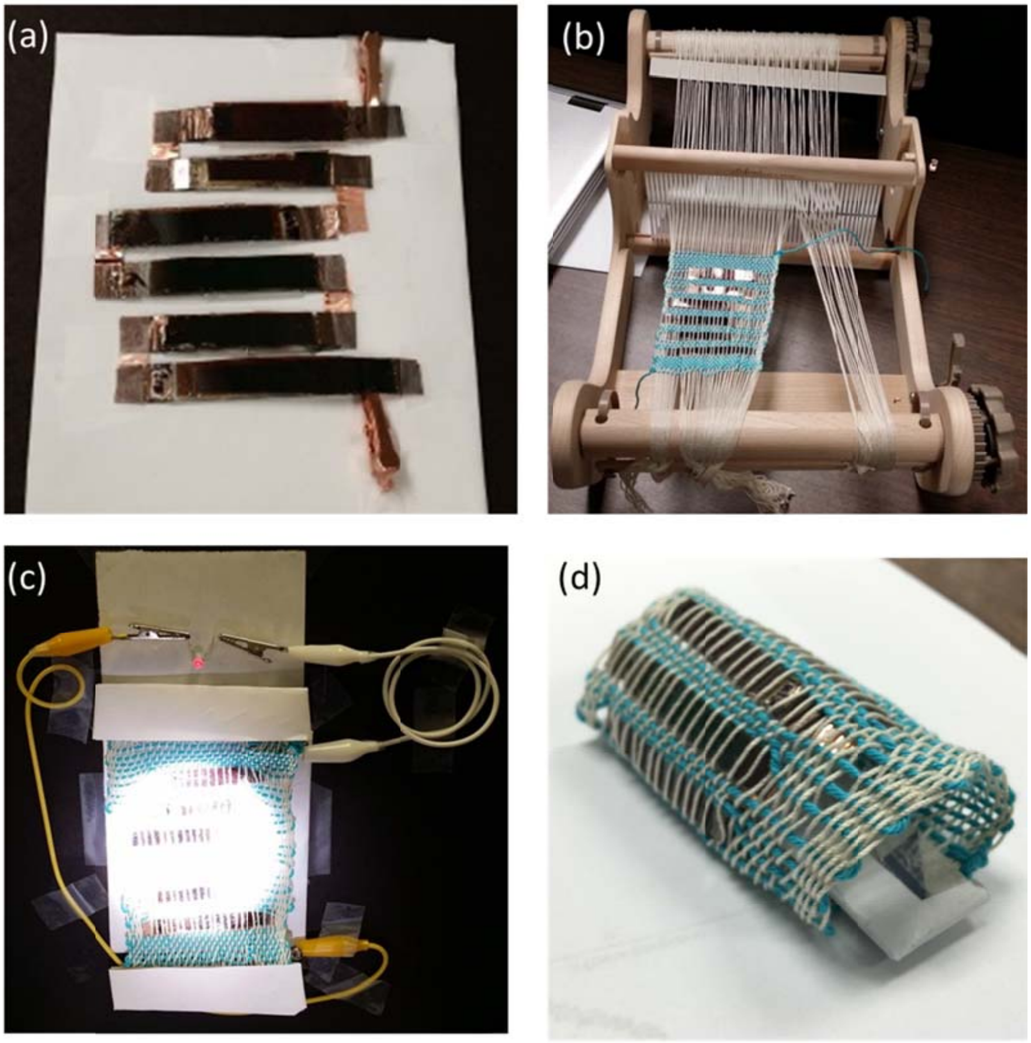

Supplementary Figure 12. (a) Photographs represent six ENHANS devices connected in series, (b) loom weaving technique to make energy-smart textile, (c) as-prepared smart textile is used to light up an LED by photo-charging with a solar simulator and (d) the fabric developed can be easily folded which is essential for portable energy applications.

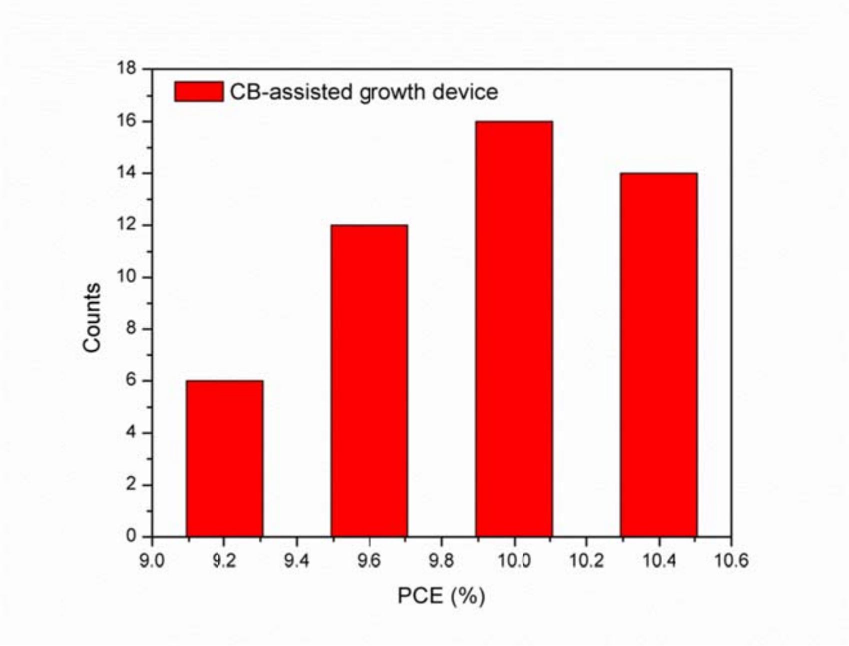

Supplementary Figure 13. Histogram of PCEs measured for 48 solar cell devices prepared by CB assisted process. All the I-V curves were measured under 100 mW cm^{-2} simulated AM 1.5G sunlight by reverse voltage scan (scan rate: 0.05 V s^{-1}).

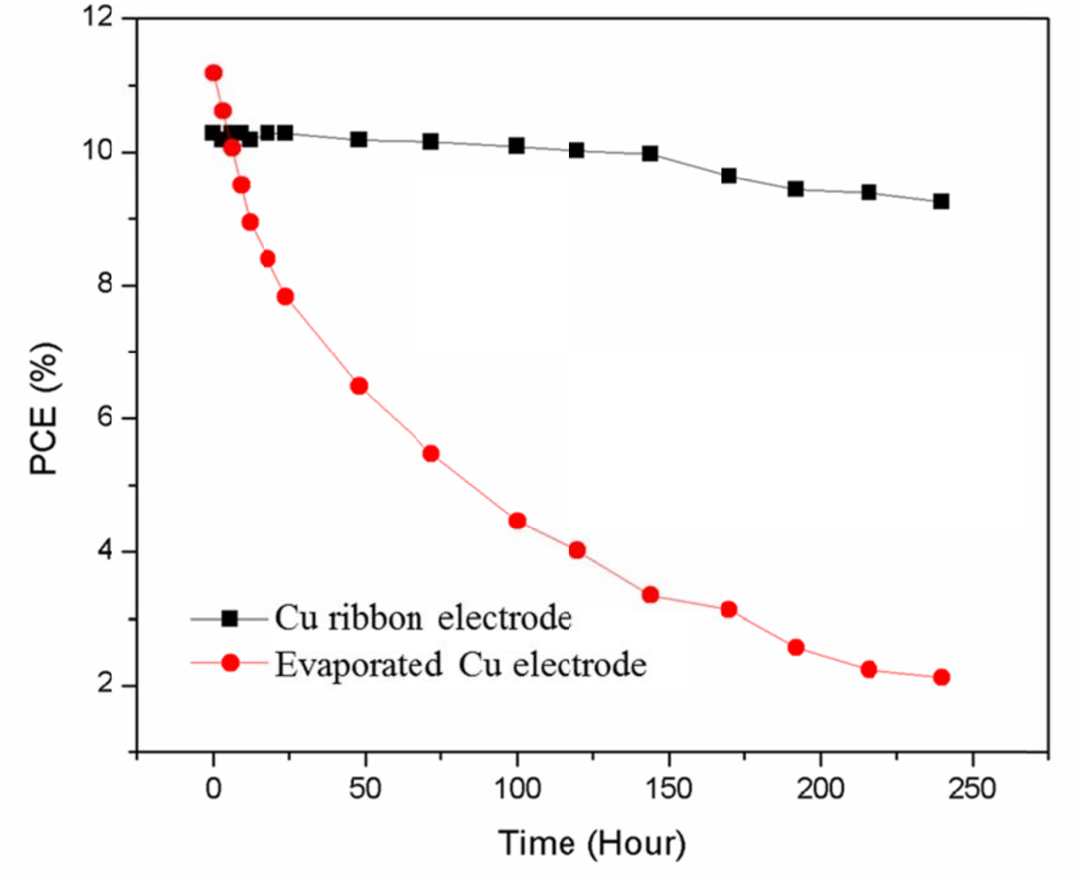

Supplementary Figure 14. The operational stability plotted with PCE vs time.

Supplementary Table 1. Gravimetric capacitance of the MnO_2 deposited electrode in three electrode configuration and device format at various scan rates. The amount (0.4 mg) of deposited MnO_2 is calculated using equation S6.

Scan rate (mV/s)	Gravimetric specific capacitance of the electrode (F g^{-1})	Gravimetric capacitance of the assembled device (F g^{-1})
5	1193.2	247.51
20	1012.5	234.36
50	863.2	211.22
100	771.68	183.09

Supplementary Table 2. A comparison of the capacitance of the supercapacitor without and with solar cell measured from the discharge time of the galvanostatic charge-discharge process.

Current density (mA cm^{-2})	Cell capacitance of the supercapacitor (mF cm^{-2})		Cell capacitance of the supercapacitor (F g^{-1})	
	Without solar cell	With solar cell	Without solar cell	With solar cell
1	48.03	37.93	288.18	227.58
2	38.76	34.72	232.56	208.32
4	32.22	33.26	193.32	199.56
8	26.99	29.01	161.94	174.06

Supplementary Table 3. A comparison of the energy density and power density of the as-prepared symmetric supercapacitor and the performance of the ENHANS ribbon under photo charging and discharging with a potentiostat.

Current density (mA cm^{-2})	Energy density						Power density					
	SSC			ENHANS			SSC			ENHANS		
	Volumetric (mWh cm^{-3})	Areal (µWh cm^{-2})	Gravimetric (µWh g^{-1})	Volumetric (mWh cm^{-3})	Areal (µWh cm^{-2})	Gravimetric (µWh g^{-1})	Volumetric (mWh cm^{-3})	Areal (µWh cm^{-2})	Gravimetric (mWh g^{-1})	Volumetric (mWh cm^{-3})	Areal (µWh cm^{-2})	Gravimetric (mWh g^{-1})
1	1.83	4.18	25.08	1.42	3.36	20.16	36.19	83.20	0.50	32.05	74.02	0.44
2	1.44	3.39	20.34	1.32	3.11	18.66	74.01	170.7	1.03	70.56	163.7	0.98
4	1.12	2.62	15.72	1.15	2.73	16.38	134.48	303.1	1.82	125.25	286.2	1.72
8	0.80	1.85	11.1	0.84	1.98	11.88	261.02	604.8	3.63	243.47	568.7	3.41

Supplementary Table 4. Reported electrodes with structured MnO_2 active materials.

Electrode	Electrolyte	Specific Capacitance	Scan rate	Year	Supplementary Ref.
$MnO_2 \cdot nH_2O$	KCl	200 F/g	5 mV/s	1999	[3]
MnO_2	NaCl	110 F/g	5 mV/s	2004	[4]
Nanostructured MnO_2	$NaSO_4$	168 F/g	5 mV/s	2006	[5]
SnO_2/MnO_2 nanowires	Na_2SO_4	637 F/g	2 mV/s	2010	[6]
TiN/MnO_2 nanotubes	KOH	123 F/g	2 mV/s	2012	[7]
C/MnO_2 nanorods	H_3PO_4	302 F/g	5 mV/s	2012	[8]
AuPd/MnO_2 nanopillar	KOH	603 F/g	5 mV/s	2013	[9]
MnO_2/ZnO/carbon-fabric	Na_2SO_4	886 F/g	2 mA/cm^2	2014	[10]
MnO_2/H-TiO2	Na_2SO_4	630 F/g	10 mV/s	2015	[11]
MnO_2 nanotubes	LiCl	1007 F/g	0.125 A/g	2015	[12]
MnO_2 nanowhiskers	KOH	1376 F/g	5 mV/s	2014	[13]
Nanocrystalline MnO_2	Li_2SO_4	1145 F/g	50 mV/s	2011	[14]
$Cu(OH)_2$/MnO_2 nanotubes	KOH	1193 F/g	5 mV/s	2016	Our work

Supplementary Methods

Preparation of the smart textile

A simple plain woven structure was used to fabricate the prototype on a hobbyist hand loom. The loom supports standard textile weaving process as found in high production machines. The weft (horizontal filling) is inserted manually. The device is interspersed with spacer cotton filaments to provide separation between devices and the flexibility to bend the fabric. The interlacement of the cotton weft filing with the longitudinal cotton warp that is drawn through the heddle provides the fabric structure with the mechanical stability needed to support the devices. The devices are inserted as a horizontal weft insert and protrude at each end. The protruding ends are used to interconnect the devices and ensure the continuity of charge flow between each segment (weft insert) of the device. The separately inserted devices are interconnected at the protruding ends. This process was done by hand, for the prototype, however a simple mechanism can be developed to automate the interconnections. The simplicity and scalability of the weaving process and the vast number of filament types that can be woven make this demonstration significant from a mass manufacture perspective.

The electrochemical performacne of the devices have been calculated by using the following equations from the previously reported literatures[1,2]:

Single electrode: Specific Capacitance (C_s) of the electrode is the capacitance per unit area of one electrode[1]. The areal specific capacitance is calculated as:

$$C_S = \frac{C_{electrode}}{A} \qquad \text{Equation S1}$$

Where A (cm^2) is the total area of the electrode.

In CV measurements, $C_{electrode}$[1] is calculated from:

$$C_{electrode} = \frac{1}{(2*\Delta V*v_0)} \int_a^b \left(\left|I_{Cu(OH)_2@AuPD@MnO_2}\right|\right) dV \qquad \text{Equation S2}$$

Where I is the current, V is the voltage, ΔV is the voltage window, v_0 is the scan rate, and a and b are voltage window boundries.

In GCD measurements, capacitance[1] can be computed as shown:

$$C_{electrode} = \frac{I \times (\Delta t_{Cu(OH)_2@AuPD@MnO_2})}{\Delta V} \qquad \text{Equation S3}$$

Where I is the discharge current, Δt is the discharge time, and ΔV is the voltage difference of discharge (obtained from the discharge curve excluding the voltage drop).

Symmetric supercapacitor device: Cell capacitance (C_{cell}) is calculated from the discharge curve of GCD measurement using the following equation:

$$C_{cell} = \frac{I \times \Delta t}{\Delta V} \qquad \text{Equation S4}$$

Where I is the discharge current, Δt is the discharge time, and ΔV is the voltage difference of discharge (obtained from the discharge curve excluding the voltage drop).

Areal specific capacitance (C_{asc}) of the device can be calculated as:

$$C_{asc} = \frac{C_{cell}}{A} \qquad \text{Equation S5}$$

Where A (cm^2) is the total area of the device.

The mass of electrodeposited MnO2 is calculated using the equation below:

$$m=\frac{I\times t\times M}{2\times e\times N_A}$$

Equation S6

where I is the current applied, t is the deposition time, M is the molecular weight of MnO2, e is the electron charge, and NA is the Avogadro's number.

Energy density and power density calculation: The energy density (E) and power density (P) can be calculated as follows:

$$E = \frac{1}{2*3600} * C_{asc} * \Delta V^2$$ Equation S7

$$P = \frac{E}{\Delta t}$$ Equation S8

Where C is the areal specific capacitance of the device, ΔV is the voltage difference of discharge and Δt is the discharge time.

Anode (charging/discharging) reaction due to physisorption and intercalation[15]:

Physisorption: $MnO_2 + A^+ + e^- \underset{discharging}{\overset{Charging}{\rightleftarrows}} MnOO^-A^+$ where A^+ is K^+ or H^+

Intercalation: $MnO_2 + A^+ + e^- \underset{discharging}{\overset{Charging}{\rightleftarrows}} MnOOA$

Cathode (Charging/Discharging):

Physisorption: $MnOO^-A^+ \underset{discharging}{\overset{Charging}{\rightleftarrows}} MnO_2 + A^+ + e^-$

Intercalation: $MnOOA \underset{discharging}{\overset{Charging}{\rightleftarrows}} MnO_2 + A^+ + e^-$

During charging process A^+ is adsorbed/intercalated on MnO_2 anode and A^+ is desorbed from the cathode as given in the above equation. During discharging process, K^+ is desorbed from the anode and adsorbed on the cathode.

Supplementary references

1 Yu, Z., Duong, B., Abbitt, D. & Thomas, J. Highly ordered MnO2 nanopillars for enhanced supercapacitor performance. *Adv. Mater.* **25**, 3302-3306 (2013).

2 Stoller, M. D. & Ruoff, R. S. Best practice methods for determining an electrode material's performance for ultracapacitors. *Energy Environ. Sci.* **3**, 1294-1301 (2010).

3 Lee, H. Y. & Goodenough, J. B. Supercapacitor behavior with KCl electrolyte. *J. Solid State Chem.* **144**, 220-223 (1999).

4 Reddy, R. N. & Reddy, R. G. Synthesis and electrochemical characterization of amorphous MnO 2 electrochemical capacitor electrode material. *J. Power Sources* **132**, 315-320 (2004).

5 Subramanian, V., Zhu, H. & Wei, B. Nanostructured MnO 2: hydrothermal synthesis and electrochemical properties as a supercapacitor electrode material. *J. Power Sources* **159**, 361-364 (2006).

6 Yan, J., Khoo, E., Sumboja, A. & Lee, P. S. Facile coating of manganese oxide on tin oxide nanowires with high-performance capacitive behavior. *ACS nano* **4**, 4247-4255 (2010).

7 Lu, X. *et al.* Stabilized TiN nanowire arrays for high-performance and flexible supercapacitors. *Nano Lett.* **12**, 5376-5381 (2012).

8 Yuan, L. *et al.* Flexible solid-state supercapacitors based on carbon nanoparticles/MnO2 nanorods hybrid structure. *Acs Nano* **6**, 656-661 (2011).

9 Yu, Z., Duong, B., Abbitt, D. & Thomas, J. Highly ordered MnO2 nanopillars for enhanced supercapacitor performance. *Adv. Mater.* **25**, 3302-3306 (2013).

10 Yang, Q. *et al.* Rationally designed hierarchical MnO 2-shell/ZnO-nanowire/carbon-fabric for high-performance supercapacitor electrodes. *J. Power Sources* **272**, 654-660 (2014).

11 Cao, X. Y. *et al.* Quantitative investigation on the effect of hydrogenation on the performance of MnO2/H-TiO2 composite electrodes for supercapacitors. *J. Mater. Chem. A* **3**, 3785-3793, doi:10.1039/C4TA06138A (2015).

12 Lu, X.-F. *et al.* High-performance supercapacitors based on MnO 2 tube-in-tube arrays. *J. Mater. Chem. A* **3**, 16560-16566 (2015).

13 Yu, Z. & Thomas, J. Energy storing electrical cables: integrating energy storage and electrical conduction. *Adv. Mater.* **26**, 4279-4285 (2014).

14 Lang, X., Hirata, A., Fujita, T. & Chen, M. Nanoporous metal/oxide hybrid electrodes for electrochemical supercapacitors. *Nat. Nanotechnol.* **6**, 232-236 (2011).

15 Toupin, M., Brousse, T. & Bélanger, D. Charge storage mechanism of MnO2 electrode used in aqueous electrochemical capacitor. *Chem. Mater.* **16**, 3184-3190 (2004).
